# Atopic Dermatitis and Water: Is There an Optimum Water Intake Level for Improving Atopic Skin?

**DOI:** 10.3390/children10020273

**Published:** 2023-01-31

**Authors:** Nikolaos Douladiris, Efstratios Vakirlis, Emilia Vassilopoulou

**Affiliations:** 1 Allergy Unit, 2nd Pediatric Clinic, University of Athens, 11527 Athens, Greece; 2 First Department of Dermatology and Venereology, School of Medicine, Aristotle University of Thessaloniki, 54124 Thessaloniki, Greece; 3 Department of Nutritional Sciences and Dietetics, International Hellenic University, 57400 Thessaloniki, Greece

**Keywords:** atopic dermatitis, atopic skin, oral hydration, water

## Abstract

Water is a vital nutrient with innumerable functions for every living cell. The functions of human skin include protection against dehydration of the body. Atopic dermatitis (AD) is a chronic pruritic inflammatory skin disease that presents with dry skin, erythematous and eczematous lesions, and lichenification. This paper discusses the question of whether extra water intake in children with AD affects skin hydration and the skin barrier function. Among the methods used to treat dry skin, topical leave-on products are the first-line treatment, intended to improve hydration and the skin barrier function. The effectiveness of adequate water intake as a measure to treat dry skin is still under debate. Normal skin hydration increases with dietary water intake, particularly in those with prior lower water consumption. Skin dryness in AD is instrumental to the itch and inflammation cycle, contributing to barrier impairment and aggravating disease severity and flares. Certain emollients provide significant hydration to AD skin, with relief of dryness and reduction in barrier impairment, disease severity, and flares. Further investigations are needed to evaluate the optimum water intake levels in children with AD, as important questions remain unanswered, namely, does oral hydration provide relief of skin dryness and reduce barrier impairment, disease severity, and flares; is there any additional benefit from using mineral or thermal spring water; or is there a need to specifically study the fluid/water intake in children with AD and food allergy (FA) restrictions?

## 1. Water for Life: Biological Functions of Water

Thales of Miletus, one of the seven Sages of Greece, referred to water as a fundamental element from which everything originated, and with which everything could be resolved [1] In the human body, water is an abundant component; in newborns, it constitutes approximately 75% of their body mass [2] During the first year of life, this proportion rapidly decreases to 60%, and remains relatively stable throughout childhood until adolescence [3]. Thereafter, hormonal changes dictate changes in the body’s composition, including a relative decrease in water content, especially in young women [4], to the range of 50–60%.

From a physiological aspect, water is a vital nutrient for every living cell, with innumerable functions. Firstly, it acts as a building material, as it constitutes approximately 76% of muscle mass [5]. It protects the maintenance of the shape and structure of human cells by creating pressure inside the cells and thus enabling them to oppose external forces. It contributes to the structure of the cell membrane by interacting with only the polar heads of phospholipids. Water adequacy ensures the structural stability of the cell membrane and ensures that the necessary molecules remain inside and the harmful molecules outside the cell. In addition, water is fundamental to the correct folding of the amino acids of proteins that function as structural elements, or as enzymes or catalysts of chemical reactions in the human body. In a similar way, water surrounds the DNA in an ordered fashion to support its characteristic double-helix configuration. In the event that this double-helix shape is lost, the DNA is unable to encode the appropriate functionality of the cells and, thus, growth, reproduction, and survival are abnormal, disrupted, or discontinued. 

Water molecules interact with each other due to their polarity, forming strong bonds known as cohesion, important in the regulation of the body’s temperature. The polarity of water leads to its interaction with other biological molecules presenting electrical asymmetry; water surrounds both their positive and negative regions, penetrates them, and dissolves them. Because of this function, water is considered to be a universal solvent and is of vital importance for the transportation of oxygen and nutrients, the optimum functioning of drugs, and for the elimination of waste products. 

## 2. Healthy Skin Hydration and Dietary Water Intake

Human skin, among other functions, protects the body against dehydration, with the outermost layer of the epidermis, unique to the skin, the stratum corneum (SC), providing a strong barrier against external, changeable, or dry environments, and controlling trans-epidermal water loss [TEWL] [6]. The SC is composed of flattened, non-nucleated keratinocytes (KCs), called corneocytes, surrounded by a complex lipid-enriched extracellular matrix [6]. Under normal skin conditions, the SC must only be impermeable enough to allow a small amount of water loss, in order to (a) hydrate the outer layers of the SC to maintain its flexibility and (b) provide water to enable the enzyme reactions that facilitate SC maturation activities. The SC uses three main mechanisms to retain water: (a) intercellular lamellar lipids, the physical conformation of which provides a tight and semi-permeable barrier to the passage of water through the tissue, (b) fully mature corneodesmosome-bound and ceramide hydrophobed corneocytes, which influence the tortuosity of the SC, and thereby the diffusion-path length of the water, and (c) intracellular and extracellular hydroscopic substances called natural moisturizing factors (NMFs) [7]. 

The regenerative capacity of skin, and maintenance of its protection against water loss among other functions, are determined by its components, the functions of which are interdependent. The water in the SC enables enzymatic activities for lipid processing, corneodesmolysis and desquamation, and the production of natural moisturizing factors (NMFs). In turn, corneodesmolysis drives the shedding of the outer layers, and the maintenance of an optimal hydration level is provided by the NMFs and permeability barrier constituted by intercellular lamellar lipids [8]. The skin’s surface contains approximately 30% of water in the SC, which increases to about 65% in the deeper layer of the epidermis, the stratum granulosum (SG) [9]. This proportion of water significantly decreases in dry skin [10], probably related to TEWL [11]. Significant for the degree of dryness is the change in the gradient of water content from the uppermost SC layer to the deeper SG [12,13]. Additionally, the sebum secreted acts together with the epidermal lipids, providing a lipid layer that enhances the maintenance of the hydration of the skin [14]. Skin barrier functionality, however, is influenced by several endogenous and environmental factors, including ethnicity, atmospheric humidity and temperature, and exposure to ultraviolet (UV) rays, chemicals, and mechanical damage [15]. 

In children, the skin is a dynamic tissue in a continuous process of maturation up to the fourth year of life, and even longer for some of its elements. This can be viewed as a period of optimization, leading to adult-like characteristics and functions [16]. The inherent physical characteristics vital for barrier function determine the length of this period and the optimum levels for everyone. This might explain, in part, why children’s skin is more vulnerable. Skin structure in children comprises smaller corneocytes, smaller keratinocytes that are more densely packed, a denser microrelief network, and more homogeneous dermal papillae, all of these constituting an epidermis that is 20% and an SC 30% thinner in relation to adult skin. Children’s skin is characterized by its lower water content, a lower concentration of NMF and surface lipids, and by higher cell proliferation and turnover; it has a low water-holding capacity, with higher absorption and desorption rates, and low TEWL. All the above mentioned factors lead to a weaker skin barrier function more prone to dryness, which under genetic and environmental influences are predisposed to diaper rash and atopic dermatitis [8].

Treatment of dry skin requires topical leave-on products’ application as the first-line action to improve the skin’s hydration and barrier function [17,18]. The effectiveness of adequate water intake as a measure to treat dry skin is still under debate [19]. In a systematic review of adult population studies conducted to explore the effect of healthy skin hydration in relation to fluid intake, despite the paucity of high-quality studies, the authors concluded that an additional intake of water may increase SC hydration, especially in individuals with lower prior water consumption [20]. Increasing the fluid intake increases the water content in the dermis, as the dermal layer can store water [21]. An effect on water content in the epidermis is also speculated, although the water content of the SC is largely determined by natural moisturizing factors, the structure of the corneocytes, and SC intercellular lipids [22].

Even less data are available on the differences in effectiveness between tap, mineral, and thermal spring water. Mac-Mary and colleagues reported that the long-term intake of mineral water improved the clinical signs of dryness and roughness in healthy subjects [23], in contrast to Williams and colleagues, who observed no significant differences in the skin’s surface morphology between the long-term drinking of mineral or tap water [24]. However, increasing one’s water intake might increase the skin’s hydration and the biomechanics by means of extensibility and the ability of the skin to return to its original state [24]. 

Considering children, to the best of our knowledge, no data have been published on the exploration of the effect of fluid intake on healthy skin’s SC hydration, at any age.

## 3. Children’s Hydration: Factors That Interfere with Optimum Hydration

The reason that children, and especially infants, are among the age groups most susceptible to dehydration, is multifactorial. Firstly, the gradual maturation of the kidneys, in combination with the high body surface-to-body mass ratio, leads to a higher rate of insensible water loss through the skin [2,25]. Infants and children present increased metabolic needs, due to their rapid rate of development, which further increases their water needs [26]. In addition, children more often suffer from infections, such as gastroenteritis, which cause diarrhea and vomiting, which induce excessive water loss [27]. The risk of dehydration is enhanced in children because they are often unaware of the sense of thirst, and therefore cannot communicate their need to drink or adequately hydrate themselves [28].

In addition to these factors, in recent years, widespread changes in eating habits, especially in Western countries, have also affected the type of fluids consumed. A trend is documented of the substitution of water intake by sugary drinks, including soft drinks, energy drinks, and packaged juices, all containing high levels of sugar, which provide empty calories, but without nutritional benefits, therefore enhancing the trends of being overweight and obese in young people [29,30]. These drinks, as processed foods, contain high amounts of additives, artificial colorings, and preservatives, which have been linked, among other health problems [31,32], to skin function abnormalities, and caffeine, a basic ingredient of several energy and soft drinks, increases diuresis, promoting dehydration [33], skin problems, such as moderate-to-severe acne, Ref. [34] and an increase in obesity rates in children [35]. In obese subjects, the skin presents dryness and higher TEWLs than in normal-weight subjects, which is related to the metabolic changes associated with obesity [36], while obese children generally tend to drink more sugary beverages, such as soft drinks, and less water [29], and appear to be more dehydrated than normal-weight children [37]. Increased body fat augments the levels of subcutaneous fat, produces skin folds, and increases surface roughness [38,39]. Consequently, several chronic systemic inflammatory skin conditions are associated with increased body weight, in both children and adults, including psoriasis [40,41,42], hidradenitis suppurativa [43], melanomas [44] and AD [45]. 

Recognizing the importance of adequate water intake, the European Food Safety Authority (EFSA) has suggested optimum amounts for children based on their age group: 800 to 1000 mL/day for infants of 6–12 months old; 1100 to 1200 mL/day for those of 2 to 3 years old; 1600 mL/day from 4 to 8 years old; 2100 mL/day for boys aged 9 to 13 years old; and 1900 mL/day for girls aged 9–13 years old. Adolescents 14 years old and above are considered as adults with respect to adequate water intake, and for moderate physical activity and non-extreme environmental temperatures, males require approximately 2500 mL/day and females approximately 2000 mL/day [46,47].

## 4. The Pathophysiology of Atopic Dermatitis and The Role of Water: A Special Consideration for Children

AD is a chronic pruritic inflammatory skin disease, presenting with dry skin, erythematous, eczematous lesions, and lichenification [48]. It is the most common inflammatory skin disease in childhood, affecting 15–20% of them [49]. Most cases of the disease arise during the first and the subsequent four years of life, during the period of optimization of the new skin barrier [50]. It more commonly affects skin sites with thinner SCs, and typically facial skin [51]. It is characterized by the interaction between skin barrier impairment and an abnormal immune response, featuring enhanced type 2 inflammation, which is particularly augmented in children [52,53]. Skin barrier impairment is characterized by a thinner epidermis, poor hydration, raised TEWL, and increased permeability to microbes, irritants, and allergens. These changes are observed in both lesional and non-lesional sites. They result from a combination of abnormal KC differentiation, less mature surface corneocytes, an increased rate of desquamation, and defective formation of ceramides and lipid lamellae, leading to altered SC homeostasis. This impairment of the skin barrier’s structures is mainly due to defective SC protein, enzyme, and lipid components [51].

SC protein components include: (a) keratin that forms pairs and filaments, which interact with the cell membrane to provide structural stability and flexibility to the KCs. The KCs, by becoming compact due to keratin crosslinking, undergo cornification to become corneocytes. Under normal conditions, upon exposure to water, the keratin-filled corneocytes swell and expand. Keratin expression is dysregulated in AD, with certain keratins being decreased or increased, corresponding to the abnormal differentiation of the KCs. (b) Filaggrin (FLG) that binds to keratin filaments in the KC, transforming KC into the less pervious corneocytes (i.e., “bricks”). FLG degradation by proteases produces natural moisturizing factors (NMFs). FLGs and NMFs are produced in a finely balanced process crucial to skin hydration and barrier function. The loss of associated mutations and reduction in the expression of FLGs are common in AD, and are associated with earlier onset, severe AD. Notably, the type 2 inflammatory environment, including changes in the interleukins IL-4, IL-13, IL-31, and IL-33, and thymic stromal lymphopoietin (TSLP) reduce FLG expression with or without FLG mutations. NMFs are composed of FLG degradation products (i.e., free amino acids, urocanic acid, and pyrrolidine carboxyl acid), which, along with urea and lactate, form sweat. NMFs retain moisture by promoting epidermal hydration through osmotic gradients that allow for the movement of water into corneocytes. NMFs also promote epidermal maturation and desquamation, contributing to normal barrier function. A decrease in the level of NMFs is associated with skin dryness and AD. Additionally, IL-4 and IL-13 in the microenvironment, by reducing FLG levels and sweat secretion, decrease the production and function of NMFs [54].

SC enzymes mainly consist of serine proteases with multiple roles. They influence SC cohesion, degrade corneodesmosome proteins during homeostatic desquamation, regulate lipid synthesis by degrading lipid enzymes, and reduce lipid secretion into the extracellular matrix. Serine protease activity is increased in lesional and non-lesional AD skin, compromising the skin’s barrier function by increasing the degradation of corneodesmosomes and extracellular lipid enzymes and reducing the production of lipids (ceramides). Protease activity is also affected by environmental influences, such as exogenous proteases from allergens (house dust mites, molds, and grass) and bacteria (*Staph aureus*), which express the same activity [54].

SC lipids are composed of approximately 47% ceramides, 24% cholesterol, 18% cholesterol esters, and 11% free fatty acids [54]. SC contains different types of ceramides, the most important of which are highly hydrophobic, very-long fatty acid chains. The lipids form densely packed layers in the central SC, becoming less densely packed and more gel-like in the outer layers. Alterations of this pattern, resulting from lipid-chain shortening and/or an altered composition of the lipids, significantly contribute to skin barrier impairment in AD. Fatty acid chains are lengthened by elongases. The expression of elongases is reduced in lesional AD, resulting in short fatty acid chains and increased skin barrier permeability. The higher the proportion of short-chain fatty acids, the higher the severity of AD. In addition, IL-4 and IL-13 inhibit the expression in KCs of elongases, and IL-4 inhibits ceramide synthesis [54]. Consequently, the main mechanisms in SC responsible for water loss are all impaired in AD, as reflected in the extension and severity of the disease expressed as skin dryness or xerosis.

Defects in proteins, such as filaggrin [55], proteases, and keratins [56,57], further increase epidermal permeability to allergens and microbes, and this is recognized as the first step in the atopic progression to the development of AD [54].

Immune system dysregulation, mainly in the expression of type 2 chemokines, such as IL-14 and IL 13, leads to the suppression of antimicrobial peptides and allergic inflammation [58,59]. IL-31 is linked to the induction of itching in patients with AD, by producing a brain-derived natriuretic peptide and coordinating chemokine release from skin cells [60,61]. IL -22 is upregulated in the skin of patients with AD with skin barrier dysfunctions and abnormal epidermal markers, such as certain keratins [62,63,64]. Vitamin D-receptor polymorphisms and the cytochrome P450 family 27 subfamily A member 1 (CYP27A1) variant is also associated with AD; CYP27A1 is involved in the metabolism of vitamin D3, which plays an important role in immune modulation [65].

Special consideration should be given to children, especially infants and toddlers, because of the complex and dynamic interrelations between an evolving disease with different pheno- and endotypes, and the concurrent events in the developing child, mainly the maturation of the skin, immune system, and overall metabolism. The study of skin barrier impairment and water loss in children should take into consideration: (a) all the aforementioned immaturities of structure and function, acting synergistically with the effect of AD structural deficiencies and inflammation on the SC, (b) the downregulation of genes, specifically lipid barrier genes that mainly account for the compromise of the skin barrier [66], and (c) the higher proportion of skin surface-to-body area in children, compared to that in adults [67].

## 5. Hydration in Children: Factors That Interfere with Optimum Hydration in Children with Atopic Dermatitis

AD is often the starting point of the allergic progression that may later lead to food allergies (FAs), allergic rhinitis, and asthma. The basic management of AD includes bathing, the application of local moisturizers, and avoidance of specific food allergens in the event of a confirmed allergic reaction. The co-existence of AD and FA is reported in approximately one third of children with AD [68]. In practice, diets involving the unnecessary, worthless, restriction of common food allergens, such as milk and eggs, are followed by more than 60% of children with AD, limiting their food variety, energy intake, and consumption of macronutrients (proteins, carbohydrates, and fats) and micronutrients (such as calcium, iron, phosphorus, B12, and riboflavin), and potentially impairing their growth [69]. The restriction of common allergenic foods is often decided upon by the caregiver with the self-perception that eczema exacerbations will be avoided, but also due to the misinterpretation of medical advice [70,71]^.^

According to the results of Engell, in an intervention study where food/energy intake was limited, there was an interdependency of water intake with food intake, as the sense of thirst was reduced when food intake was reduced [72]. Taking into consideration that in children with AD, unreasonable food restrictions may limit food intake [69,73,74,75], it is possible that the intake of water and other liquids might be diminished in parallel, constituting an additional risk of dehydration.

Although children with AD often have a shorter stature [76,77,78,79], and lower weight in infancy and early childhood, in some cohorts they are reported as overweight in comparison to their healthy peers [80,81]. In addition, infants whose mothers were obese or overweight before pregnancy appear to have a higher risk of developing AD [82]. Children who are overweight or obese experience more severe symptoms of AD [80], and it is suggested that both the incidence and severity of AD in children with an increased body mass index (BMI) are due to the immunomodulating properties of adipokines, such as leptin [83] and ghrelin [84].

Children with AD often avoid dairy products and limit their choices in consuming specific food groups, such as fruits and vegetables, due to the perception of their parents that they trigger AD symptoms. Fresh fruits and vegetables, and their fresh juices, are an important source of hydration and are recommended for children as an alternative source of water, in efforts to achieve appropriate hydration [85]. Specific fruits, such as citrus fruits, strawberries, and kiwi, but also vegetables, such as tomato and spinach, including their fresh juices, are among the most common “scapegoats” for AD exacerbations [70]. However, these items constitute an important source of vitamin C, a component with a strategic role in the ceramide production in KCs. The immunomodulating mechanism involves the alteration of the mechanism of ceramide metabolic-related enzymes that improve the overall epidermal barrier function [86]. On the other hand, prepacked juices and other popular products contain food additives that further increase skin permeability in children with AD, resulting in further exacerbations of atopic skin symptoms [87].

As described above, among the foods that are more frequently, and without reason, eliminated from the diet of children with AD are milk and dairy products, which results in an overall reduction in their daily liquid intake [70,87]. Milk has been proposed as an effective rehydration beverage for children, even after exercise [88]. The study by Hon and colleagues provided evidence that children without sensitization to milk had less severe AD outcomes when they consumed milk [89]. In that study, children with AD tended to substitute milk with herbals and soymilk, and they presented reduced skin hydration in comparison to children without AD [89].

A milk-elimination diet significantly reduces the daily intake of calcium, which is an important nutrient for the maintenance of the epidermal barrier, as it is involved in KC differentiation. The differentiation of KCs is a crucial process in the formation of the various different layers of the skin, including the SC, which functions as a barrier for diminishing water loss [52].

Another issue in relation to milk avoidance and skin health is that fortified milk is among the most effective sources of vitamin D [90]. Milk intake has been positively associated with the maintenance of normal vitamin D levels [90,91], and it is associated with a wide spectrum of skin diseases involving the immune system, including psoriasis, melanoma, alopecia, and AD [92]. It is recognized that the most important source of vitamin D in the human body is sunlight: whole-body exposure to UVB radiation, inducing the light-pink color of the minimal erythema dose, for 15–20 min is capable of inducing the production of 250 μg vitamin D [93]. On the other hand, exposure to UVB radiation has been linked with acute and chronic harmful health effects, such as skin aging and cancer [94], dictating that exposure to the sunlight must be undertaken with care [95]. The contradiction between the need for vitamin D and the adverse health effects of Sun UVB radiation have led to the vitamin D-deficiency epidemic [96]. Several skin conditions have been linked with vitamin D deficiency, including poor wound healing, psoriasis, acne and rosacea, and hair loss [93]. The research interest has also focused on AD, where vitamin D supplements have been used as a preventive measure for AD and atopy in early life [97] and for reducing the severity of the disease [98,99]. An interesting outcome that needs to be researched further is the observation of a Danish group that measured the changes in the bioavailability of vitamin D when the diet was supplemented with water, milk, or juice. They noted that water was as effective as milk in facilitating vitamin D absorption [100]. This result could be justified by the changes in intestinal permeability when water intake is increased, leading to an increased absorption of vitamin D. This assumption is supported further by a study on critically ill patients, where it was observed that vitamin D levels are dependent on intestinal permeability [101]. Intestinal permeability is affected by the quality of water, according to Dupuy and colleagues, who provided per os low-salt water in patients with AD with increased intestinal permeability, and observed an associated decrease [95]. It thus appears that adequate water ingestion facilitates the absorption of several nutrients, including vitamin D, but this warrants further investigation.

## 6. Treatment of Atopic Dermatitis

The management of AD focuses on three main targets:

(a) Avoidance of aggravating factors;
(b) Treatment of the underlying inflammation;
(c) Restoration of the skin barrier as the essence of management.

The avoidance of aggravating factors should improve the patient’s quality of life. This requires a detailed diary of everyday activities and environments to reveal the factors that can be avoided or managed to significantly improve the patient’s life with the disease [102]. 

The treatment of underlying inflammation, both topically and systemically, is entering a new era of targeted and stratified medicine, with new and advanced therapeutic choices for all ages. The successful control of the inflammation will provide definitive control of AD and of a major part of skin barrier impairment [102].

However, the restoration of the skin’s barrier, focused on the restoration of the SC, will continue to be the essence of management of AD, because (a) most cases are of mild-to-moderate severity, and will require reactive, intermittent, anti-inflammatory treatment; (b) most of the cases of AD occur in infants and toddlers with mild-to-moderate disease, and there are certain restrictions to the use of anti-inflammatory treatment; and, mainly, (c) the initiating step of the disease is the genetically determined SC defect that will necessitate its own individual treatment [102]. 

It is known that the prolonged application of a water-holding substance on healthy skin increases the water content throughout the SC. This water is then gradually released from the upper SC after the discontinuation of the hydration procedure. This indicates an important role of applicable water-holding substances, such as certain moisturizers, in the regulation of SC water content, but also the restricted time of their action [103]. Additionally, in blinded, randomized trials on healthy skin, it can be observed that certain emollients could improve water gradients and SC hydration. Those emollients include ingredients that can substitute or increase epidermal lipogenesis and SC barrier function [104]. Various emollients, with different hydration capacities, are used for the treatment of AD, each with a different, but restricted, duration of action, after which reapplication is needed. Based on such data, the guidelines recommend that the hydration of the skin is usually maintained by the application, at least twice daily, of emollients with a hydrophilic base, such as glycerol or urea [102].

Consequently, basic, topical emollient therapy constitutes the essence of every treatment regimen of AD. Emollients should, and usually do, contain (a) an occludent (to reduce evaporation), such as lipids, which, ideally, will replace in part defective skin lipid function, and (b) a humectant or moisturizer to promote the hydration of the SC, such as glycerol or urea. This, ideally, will replace in part the defective NMF’s function, restore the water-holding capacity, and reduce skin dryness [102]. A Cochrane review comparing emollients containing moisturizers with those containing no moisturizers determined that the former was better at reducing investigator-reported severity and was associated with fewer flares and less topical corticosteroid use [105]. These results highlight the importance of preserving hydration in the SC in the management of AD. How does the water in the SC plays a critical role in skin homeostasis? Depending on their mobility that is determined by their hydrogen bonds and space limitations, SC water molecules “move” into three levels: (a) “bound” (least mobile) molecules that are directly bonded with SC molecules, (b) intermediately mobile molecules that form hydrogen bonds with “bound” water molecules, forming a “loose cloud” around the binding site, and (c) the most mobile molecules that can diffuse freely, constantly forming and breaking bonds with surrounding water molecules [106]. These weakly defined “states” constitute a continuum of bound states and provide a perspective on the mobility of water molecules in the SC [107]. The bound and most mobile water molecules decrease toward the skin’s surface, while the intermediate group increases. These changes are subtle but statistically significant, and are constant at different adult ages and body sites, implying a controlled mechanism to define them [107]. They appear to be in accordance with: (a) a gradual increase in NMF concentrations toward the SC surface, indicative of proteolytic processes along the SC exposing pockets of bound water molecules, increasing their mobility, and (b) a decrease in lipids toward the outer SC, indicative of diminished processes along the lipid headgroups bound in water, increasing their mobility. These observations provide important information for the dynamic equilibrium of water molecules in the SC of healthy skin and their contribution to skin hydration. More importantly, they present the question of how these patterns differ in children and in deficit disorders of barrier elements (i.e., NMFs and lipids), such as AD [107]. Is there an increase in most mobile water molecules in the SC in AD, instead of the intermediate group, due to deficits in lipids and NMFs, and consequently increased water loss and skin dryness? Additionally, if so, would an increase in water intake result in an increase in “bound” and intermediate water molecules that would counterbalance, in part, the loss of mobile water molecules, and thus improve the skin’s dryness?

## 7. Dietary Water Intake and Atopic Dermatitis

The data on the overall water intake, quality of water, and hydration status in patients with AD are limited. Three studies analyzed the relationship between water hardness and chlorine levels, and the prevalence of AD. First McNally and colleagues showed a correlation between water hardness and chlorine levels, and an increased prevalence of AD in primary-school children [108]. These results were later confirmed by Miyake and colleagues in a cohort study involving children aged 6–7 years, and they speculated that water rich in calcium and magnesium might be involved in the pathophysiological mechanisms of AD [109]. These results were replicated in a Spanish cohort of 6–7- and 13–14-year-old children by Arnedo-Pena and colleagues; however, a significant relationship was observed only in the younger age group [110]. None of these studies investigated the effect of consuming this type of water on AD incidence or exacerbation.

Kimata and colleagues reported that patients with AD presented an improvement in skin symptoms, with the control of the production of inflammatory/atopy cytokines when they drank mineral deep-sea water rich in calcium and other minerals, while distilled water failed to provide any benefits [111]; moreover, a 100 mL increase in overall fluid intake was associated with a slight decrease in the sebum content, but had no effect on hydration [111]. Hataguchi and colleagues conducted an intervention program on a group of 33 adult patients with AD. The patients drank 500 mL of deep-sea water with a high-magnesium and low-sodium chloride (NaCl) content for 6 months, and an evaluation of the clinical symptoms was performed using different scoring systems for inflammation, lichenification, and cracking, at different body sites. Additionally, the researchers measured the levels of various essential and toxic minerals [112]. They reported an improvement in skin symptoms in 27/33 patients with AD, and concluded that the deep-sea water, which facilitated the elimination of toxic metals, specifically mercury and lead, and increased the levels of the antioxidant selenium, may play a role in the treatment of AD [112]. 

Considering thermal spring water, it is known that there are certain differences in the mineral compositions in different springs, which is considered important for the treatment of normal skin and certain dermatoses [113]. Patients are also encouraged, during their spa course, to drink thermal spring water as part of the program [95]. However, in contrast to the documentation of the curative effects of certain thermal spring waters on the skin’s structure and function after bathing, there are no data on any possible benefits, qualitative or quantitative, to the skin after drinking the same thermal spring waters. 

## 8. Questions to Answer—Research Needs

The aim of this paper was to propose the question of whether additional water intake affects the hydration of the skin and skin barrier function in children with AD.

Childhood, and especially infancy, is the most vulnerable period for dehydration, due to ongoing developmental characteristics during this time. Children in Westernized-lifestyle communities are more prone to dehydration because of their everyday drinking, eating, and socializing habits, which do not promote water intake according to the recommended levels. Children suffering from AD are likely to be even more prone to dehydration, due to additional, necessary, more often unnecessary, restriction diets and restricted social activities.

In the adult population, normal skin hydration increases with dietary water intake, particularly in those with prior lower water consumption levels. Normal skin hydration in children is in a more vulnerable state, and for those with prior lower water consumption levels, it may be easiest to rebalance this with an increased water intake. 

AD is characterized by skin dryness due to defective stratum corneum protein and enzyme and lipid components. AD in children is characterized by increased proneness to dryness due to skin immaturity and a high body surface-to-body mass ratio, in addition to the abovementioned deficits. Skin dryness in AD significantly contributes to the itching and inflammation cycle, aggravating barrier impairment and disease severity and flares.

Certain emollients provide significant skin hydration effects and the relief of skin dryness, reducing barrier impairment, itching, disease severity, and flares in AD. However, this is a transient relief that necessitates repeated application, almost a lifelong one.

Based on these data, certain, simple research questions arise that encourage further investigations:

Will increased water intake increase the hydration of children’s skin in AD, offering at least partial relief for skin dryness and reduction in barrier impairment, disease severity, and flares? 

Could mineral or thermal spring water intake provide any additional benefits for childhood AD, with relief of skin dryness and a reduction in barrier impairment, disease severity, and flares? 

Is there a need to specifically study fluid/water intake levels in children with AD and FA restrictions? 

## References

[1] Riveros-Perez E., Riveros R. (2017). Water in the human body: An anesthesiologist’s perspective on the connection between physicochemical properties of water and physiologic relevance. Ann. Med. Surg..

[2] Lindower J.B. (2017). Water balance in the fetus and neonate. Semin. Fetal Neonatal Med..

[3] Novak L.P. (1989). Changes in total body water during adolescent growth. Hum. Biol..

[4] Bottin J.H., Morin C., Guelinckx I., Perrier E.T. (2019). Hydration in Children: What Do We Know and Why Does it Matter?. Ann. Nutr. Metab..

[5] Lorenzo I., Serra-Prat M., Yébenes J.C. (2019). The Role of Water Homeostasis in Muscle Function and Frailty: A Review. Nutrients.

[6] Akdeniz M., Gabriel S., Lichterfeld-Kottner A., Blume-Peytavi U., Kottner J. (2018). Transepidermal water loss in healthy adults: A systematic review and meta-analysis update. Br. J. Dermatol..

[7] Rodrigues L.M., Palma L., Marques L.T., Varela J.B. (2015). Dietary water affects human skin hydration and biomechanics. Clin. Cosmet. Investig. Dermatol..

[8] Stamatas G.N., Nikolovski J., Mack M.C., Kollias N. (2010). Infant skin physiology and development during the first years of life: A review of recent findings based on in vivo studies. Int. J. Cosmet. Sci..

[9] Caspers P.J., Lucassen G.W., Carter E.A., Bruining H.A., Puppels G.J. (2001). In Vivo Confocal Raman Microspectroscopy of the Skin: Noninvasive Determination of Molecular Concentration Proles. J. Investig. Dermatol..

[10] Mccallion R., Li A., Po W. (1993). Dry and Photo-Aged Skin: Manifestations and Management. J. Clin. Pharm. Ther..

[11] Kottner J., Lichterfeld A., Blume-Peytavi U. (2013). Transepidermal water loss in young and aged healthy humans: A systematic review and meta-analysis. Arch. Dermatol. Res..

[12] Igaki M., Higashi T., Hamamoto S., Kodama S., Naito S., Tokuhara S. (2013). A study of the behavior and mechanism of thermal conduction in the skin under moist and dry heat conditions. Ski. Res. Technol..

[13] Rawlings A.V., Harding C.R. (2004). Moisturization and Skin Barrier Function. Dermatol. Ther..

[14] Boer M., Duchnik E., Maleszka R., Marchlewicz M. (2016). Structural and biophysical characteristics of human skin in maintaining proper epidermal barrier function. Postepy Dermatol. Alergol..

[15] Lee M., Jung Y., Kim E., Lee H.K. (2016). Comparison of skin properties in individuals living in cities at two different altitudes: An investigation of the environmental effect on skin. J. Cosmet. Dermatol..

[16] Mack M.C., Chu M.R., Tierney N.K., Ruvolo E., Stamatas G.N., Kollias N., Bhagat K., Ma L., Martin K.M. (2016). Water-Holding and Transport Properties of Skin Stratum Corneum of Infants and Toddlers Are Different from Those of Adults: Studies in Three Geographical Regions and Four Ethnic Groups. Pediatr. Dermatol..

[17] Lodén M. (2016). Treatments Improving Skin Barrier Function. Curr. Probl. Dermatol..

[18] Kottner J., Surber C. (2016). Skin care in nursing: A critical discussion of nursing practice and research. Int. J. Nurs. Stud..

[19] Wolf R., Wolf D., Rudikoff D., Parish L.C. (2010). Nutrition and water: Drinking eight glasses of water a day ensures proper skin hydration—Myth or reality?. Clin. Dermatol..

[20] Akdeniz M., Tomova-Simitchieva T., Dobos G., Blume-Peytavi U., Kottner J. (2018). Does dietary fluid intake affect skin hydration in healthy humans? A systematic literature review. Ski. Res. Technol..

[21] Eisenbeiss C., Welzel J., Eichler W., Klotz K. (2001). Influence of Body Water Distribution on Skin Thickness: Measurements Using High-Frequency Ultrasound. Br. J. Dermatol..

[22] Berardescal E. (1997). EEMCO Guidance for the Assessment of Stratum Comeurn Hydration: Electrical Methods European Group for Efficacy Measurements on Cosmetics and Other Topical Products (EEMC0). Ski. Res. Technol..

[23] Mac-Mary S., Creidi P., Marsaut D., Courderot-Masuyer C., Cochet V., Gharbi T., Guidicelli-Arranz D., Tondu F., Humbert P. (2006). Assessment of effects of an additional dietary natural mineral water uptake on skin hydration in healthy subjects by dynamic barrier function measurements and clinic scoring. Ski. Res. Technol..

[24] Williams S., Krueger N., Davids M., Kraus D., Kerscher M. (2007). Effect of fluid intake on skin physiology: Distinct differences between drinking mineral water and tap water. Int. J. Cosmet. Sci..

[25] Manz F. (2007). Hydration in Children. J. Am. Coll. Nutr..

[26] Roy M., Vega U.A. (2022). Pediatric Dehydration.

[27] Florez I.D., Niño-Serna L.F., Beltrán-Arroyave C.P. (2020). Acute Infectious Diarrhea and Gastroenteritis in Children. Curr. Infect. Dis. Rep..

[28] D’Anci K.E., Constant F., Rosenberg I.H. (2006). Hydration and Cognitive Function in Children. Nutr. Rev..

[29] Franse C.B., Wang L., Constant F., Fries L.R., Raat H. (2019). Factors associated with water consumption among children: A systematic review. Int. J. Behav. Nutr. Phys. Act..

[30] Mantziki K., Renders C.M., Seidell J.C. (2017). Water Consumption in European Children: Associations with Intake of Fruit Juices, Soft Drinks and Related Parenting Practices. Int. J. Environ. Res. Public Health.

[31] Silva M.M., Reboredo F.H., Lidon F.C. (2022). Food Colour Additives: A Synoptical Overview on Their Chemical Properties, Applications in Food Products, and Health Side Effects. Foods.

[32] Kregiel D. (2015). Health Safety of Soft Drinks: Contents, Containers, and Microorganisms. BioMed Res. Int..

[33] Hew-Butler T., Verbalis J.G., Noakes T.D. (2006). Updated Fluid Recommendation: Position Statement from the International Marathon Medical Directors Association (IMMDA). Clin. J. Sport Med..

[34] Huang S.-L., Pan W.-H. (2001). Dietary fats and asthma in teenagers: Analyses of the first Nutrition and Health Survey in Taiwan (NAHSIT). Clin. Exp. Allergy.

[35] Abbasalizad Farhangi M., Mohammadi Tofigh A., Jahangiri L., Nikniaz Z., Nikniaz L. (2022). Sugar-Sweetened Beverages Intake and the Risk of Obesity in Children: An Updated Systematic Review and Dose–Response Meta-Analysis. Pediatr. Obes..

[36] Guida B., Nino M., Perrino N., Laccetti R., Trio R., Labella S., Balato N. (2010). The impact of obesity on skin disease and epidermal permeability barrier status. J. Eur. Acad. Dermatol. Venereol..

[37] Maffeis C., Tommasi M., Tomasselli F., Spinelli J., Fornari E., Scattolo N., Marigliano M., Morandi A. (2015). Fluid intake and hydration status in obese vs normal weight children. Eur. J. Clin. Nutr..

[38] Darlenski R., Mihaylova V., Handjieva-Darlenska T. (2022). The Link Between Obesity and the Skin. Front. Nutr..

[39] Mori S., Shiraishi A., Epplen K., Butcher D., Murase D., Yasuda Y., Murase T. (2017). Characterization of skin function associated with obesity and specific correlation to local/systemic parameters in American women. Lipids Health Dis..

[40] Onsun N., Akaslan T., Sallahoglu K., Gülcan A.S., Bulut H., Yabacı A. (2022). Effects of TNF inhibitors and an IL12/23 inhibitor on changes in body weight and adipokine levels in psoriasis patients: A 48-week comparative study. J. Dermatol. Treat..

[41] Graier T., Weger W., Sator P.-G., Salmhofer W., Gruber B., Jonak C., Kölli C., Schütz-Bergmayr M., Vujic I., Ratzinger G. (2021). Effectiveness and clinical predictors of drug survival in psoriasis patients receiving apremilast: A registry analysis. JAAD Int..

[42] Zachariae C., Skov L. (2020). Obesity as a risk factor for psoriasis. J. Eur. Acad. Dermatol. Venereol..

[43] Garcovich S., Fania L., Caposiena D., Giovanardi G., Chiricozzi A., De Simone C., Tartaglia C., Ciccone D., Bianchi L., Abeni D. (2021). Pediatric Hidradenitis Suppurativa: A Cross-Sectional Study on Clinical Features and Treatment Approaches. J. Cutan. Med. Surg..

[44] Pellegrini M., D’Eusebio C., Ponzo V., Tonella L., Finocchiaro C., Fierro M.T., Quaglino P., Bo S. (2021). Nutritional Interventions for Patients with Melanoma: From Prevention to Therapy—An Update. Nutrients.

[45] Hirt P.A., Castillo D.E., Yosipovitch G., Keri J.E. (2019). Skin changes in the obese patient. J. Am. Acad. Dermatol..

[46] Agostoni C., Bresson J.L., Fairweather-Tait S. (2010). EFSA Panel on Dietetic Products, Nutrition, Allergies, Scientific Opinion on Dietary Reference Values for water. EFSA J..

[47] European Food Safety Authority (2010). Outcome of the Public consultation on the Draft Opinion of the Scientific Panel on Dietetic Products, Nutrition, and Allergies (NDA) on establishing Dietary Reference Values for water. EFSA J..

[48] Kolb L., Ferrer-Bruker S.J. (2022). Atopic Dermatitis.

[49] Asher I., Montefort S., Björkstén B., Lai C.K.W., Strachan D.P., Weiland S.K., Williams H., Phase I. (2006). Worldwide Time Trends in the Prevalence of Symptoms of Asthma, Allergic Rhinoconjunctivitis, and Eczema in Childhood: ISAAC Phases One and Three Repeat Multicountry Cross-Sectional Surveys. Lancet.

[50] Akdis C.A., Akdis M., Bieber T., Bindslev-Jensen C., Boguniewicz M., Eigenmann P., Hamid Q., Kapp A., Leung D.Y., Lipozencic J. (2006). Diagnosis and treatment of atopic dermatitis in children and adults: European Academy of Allergology and Clinical Immunology/American Academy of Allergy, Asthma and Immunology/PRACTALL Consensus Report. J. Allergy Clin. Immunol..

[51] Danby S.G. (2016). Biological Variation in Skin Barrier Function: From A (Atopic Dermatitis) to X (Xerosis). Curr. Probl. Dermatol..

[52] Yang G., Seok J.K., Kang H.C., Cho Y.-Y., Lee H.S., Lee J.Y. (2020). Skin Barrier Abnormalities and Immune Dysfunction in Atopic Dermatitis. Int. J. Mol. Sci..

[53] Esaki H., Czarnowicki T., Gonzalez J., Oliva M., Talasila S., Haugh I., Rodriguez G., Becker L., Krueger J.G., Guttman-Yassky E. (2016). Accelerated T-cell activation and differentiation of polar subsets characterizes early atopic dermatitis development. J. Allergy Clin. Immunol..

[54] Beck L.A., Cork M.J., Amagai M., De Benedetto A., Kabashima K., Hamilton J.D., Rossi A.B. (2022). Type 2 Inflammation Contributes to Skin Barrier Dysfunction in Atopic Dermatitis. JID Innov..

[55] Moosbrugger-Martinz V., Leprince C., Méchin M.-C., Simon M., Blunder S., Gruber R., Dubrac S. (2022). Revisiting the Roles of Filaggrin in Atopic Dermatitis. Int. J. Mol. Sci..

[56] Chieosilapatham P., Kiatsurayanon C., Umehara Y., Trujillo-Paez J.V., Peng G., Yue H., Nguyen L.T.H., Niyonsaba F. (2021). Keratinocytes: Innate immune cells in atopic dermatitis. Clin. Exp. Immunol..

[57] Gallegos-Alcalá P., Jiménez M., Cervantes-García D., Salinas E. (2021). The Keratinocyte as a Crucial Cell in the Predisposition, Onset, Progression, Therapy and Study of the Atopic Dermatitis. Int. J. Mol. Sci..

[58] Fania L., Moretta G., Antonelli F., Scala E., Abeni D., Albanesi C., Madonna S. (2022). Multiple Roles for Cytokines in Atopic Dermatitis: From Pathogenic Mediators to Endotype-Specific Biomarkers to Therapeutic Targets. Int. J. Mol. Sci..

[59] Turchin I., Bourcier M. (2022). The Role of Interleukins in the Pathogenesis of Dermatological Immune-Mediated Diseases. Adv. Ther..

[60] Borgia F., Custurone P., Pomi F.L., Cordiano R., Alessandrello C., Gangemi S. (2022). IL-31: State of the Art for an Inflammation-Oriented Interleukin. Int. J. Mol. Sci..

[61] Furue M. (2020). Regulation of Filaggrin, Loricrin, and Involucrin by IL-4, IL-13, IL-17A, IL-22, AHR, and NRF2: Pathogenic Implications in Atopic Dermatitis. Int. J. Mol. Sci..

[62] Prignano F., Donetti E. (2022). Looking at Interleukin-22 from a New Dermatological Perspective: From Epidermal Homeostasis to Its Role in Chronic Skin Diseases. Dermatology.

[63] Uchiyama A., Fujiwara C., Inoue Y., Ishikawa M., Motegi S.I. (2022). Possible Suppressive Effects of Baricitinib on Serum IL-22 Levels in Atopic Dermatitis. J. Dermatol. Sci..

[64] Sugaya M. (2020). The Role of Th17-Related Cytokines in Atopic Dermatitis. Int. J. Mol. Sci..

[65] Adami G., Fassio A. (2021). OPEN ACCESS Vitamin D Supplementation: Better Daily or by Bolus?. Vitam. D-Updates.

[66] Brunner P.M., Israel A., Zhang N., Leonard A., Wen H.-C., Huynh T., Tran G., Lyon S., Rodriguez G., Immaneni S. (2018). Early-onset pediatric atopic dermatitis is characterized by TH2/TH17/TH22-centered inflammation and lipid alterations. J. Allergy Clin. Immunol..

[67] Fakoya A.O.J., Omenyi P., Anthony P., Anthony F., Etti P., Otohinoyi D.A., Olunu E. (2018). Stevens—Johnson Syndrome and Toxic Epidermal Necrolysis; Extensive Review of Reports of Drug-Induced Etiologies, and Possible Therapeutic Modalities. Open Access Maced. J. Med. Sci..

[68] Youm S., Lee E., Lee J. (2021). Environmental and dietary factors to be checked for treatment of atopic dermatitis in rural children. Clin. Exp. Pediatr..

[69] Lim H., Song K., Kim R., Sim J., Park E., Ahn K., Kim J., Han Y. (2013). Nutrient Intake and Food Restriction in Children with Atopic Dermatitis. Clin. Nutr. Res..

[70] Nosrati A., Afifi L., Danesh M.J., Lee K., Yan D., Beroukhim K., Ahn R., Liao W. (2017). Dietary modifications in atopic dermatitis: Patient-reported outcomes. J. Dermatol. Treat..

[71] Jeon Y.H. (2022). Dietary restriction misconceptions and food allergy education in children with atopic dermatitis. Clin. Exp. Pediatr..

[72] Engell D. (1988). Interdependency of Food and Water Intake in Humans. Appetite.

[73] Low D., Jamil A., Nor N., Ibrahim S.B.K., Poh B.K. (2019). Food restriction, nutrition status, and growth in toddlers with atopic dermatitis. Pediatr. Dermatol..

[74] Cui H.S., Ahn I.S., Byun Y.S., Yang Y.S., Kim J.H., Chung B.Y., Kim H.O., Park C.W. (2014). Dietary Pattern and Nutrient Intake of Korean Children with Atopic Dermatitis. Ann. Dermatol..

[75] Park M.K., Park K.Y., Li K., Seo S.J., Hong C.K. (2013). The Short Stature in Atopic Dermatitis Patients: Are Atopic Children Really Small for Their Age?. Ann. Dermatol..

[76] Ellison J., Patel L., Kecojevic T., Foster P., David T., Clayton P. (2006). Pattern of growth and adiposity from infancy to adulthood in atopic dermatitis. Br. J. Dermatol..

[77] Kristmundsdottir F., David T.J. (1987). Growth Impairment in Children with Atopic Eczema. J. R. Soc. Med..

[78] Massarano A.A., Hollis S., Devlin J., David T.J. (1993). Growth in atopic eczema. Arch. Dis. Child..

[79] Palit A., Handa S., Kumar Bhalla A., Kumar B. (2007). A Mixed Longitudinal Study of Physical Growth in Children with Atopic A Mixed Longitudinal Study of Physical Growth in Children with Atopic Dermatitis Dermatitis. Indian J. Dermatol. Venereol. Leprol..

[80] Zhang A., Silverberg J.I. (2015). Association of atopic dermatitis with being overweight and obese: A systematic review and metaanalysis. J. Am. Acad. Dermatol..

[81] Nicholas M.N., Keown-Stoneman C.D.G., Maguire J.L., Drucker A.M. (2022). Association Between Atopic Dermatitis and Height, Body Mass Index, and Weight in Children. JAMA Dermatol..

[82] Wei X., Huang P., Gao C., Shen S., Tu S., Guo Y., Zhang L., Lu M., Lu J., Wang C.C. (2022). Associations of maternal weight status with the risk of offspring atopic dermatitis and wheezing by 1 year of age. Pediatr. Allergy Immunol..

[83] Jiménez-Cortegana C., Ortiz-García G., Serrano A., Moreno-Ramírez D., Sánchez-Margalet V. (2021). Possible Role of Leptin in Atopic Dermatitis: A Literature Review. Biomolecules.

[84] Jaworek A.K., Szepietowski J.C., Szafraniec K., Jaworek M., Hałubiec P., Wojas-Pelc A., Pokorski M. (2020). Adipokines as Biomarkers of Atopic Dermatitis in Adults. J. Clin. Med..

[85] Lai J.S., Hiles S., Bisquera A., Hure A.J., McEvoy M., Attia J. (2013). A systematic review and meta-analysis of dietary patterns and depression in community-dwelling adults. Am. J. Clin. Nutr..

[86] Kim K.P., Shin K.-O., Park K., Yun H.J., Mann S., Lee Y.M., Cho Y. (2015). Vitamin C Stimulates Epidermal Ceramide Production by Regulating Its Metabolic Enzymes. Biomol. Ther..

[87] Anıl H., Harmanci K. (2020). Evaluation of contact sensitivity to food additives in children with atopic dermatitis. Adv. Dermatol. Allergol..

[88] Volterman K.A., Obeid J., Wilk B., Timmons B.W. (2014). Effect of milk consumption on rehydration in youth following exercise in the heat. Appl. Physiol. Nutr. Metab..

[89] Hon K.L., Tsang Y.C., Poon T.C.W., Pong N.H.H., Luk N.M., Leung T.N.H., Chow C.M. (2015). Dairy and nondairy beverage consumption for childhood atopic eczema: What health advice to give?. Clin. Exp. Dermatol..

[90] Torres-Gonzalez M., Cifelli C.J., Agarwal S., Fulgoni V.L. (2020). Association of Milk Consumption and Vitamin D Status in the US Population by Ethnicity: NHANES 2001–2010 Analysis. Nutrients.

[91] Itkonen S.T., Erkkola M., Lamberg-Allardt C.J.E. (2018). Vitamin D Fortification of Fluid Milk Products and Their Contribution to Vitamin D Intake and Vitamin D Status in Observational Studies—A Review. Nutrients.

[92] Navarro-Triviño F., Arias-Santiago S., Gilaberte-Calzada Y. (2019). Vitamina D y la piel. Una revisión para dermatólogos. Actas Dermo-Sifiliográficas.

[93] Mostafa W.Z., Hegazy R.A. (2014). Vitamin D and the skin: Focus on a complex relationship: A review. J. Adv. Res..

[94] Knuschke P. (2021). Sun Exposure and Vitamin D. Curr. Probl. Dermatol..

[95] Dupuy P., Cassé M., André F., Dhivert-Donnadieu H., Pinton J., Hernandez-Pion C. (1999). Low-Salt Water Reduces Intestinal Permeability in Atopic Patients. Dermatology.

[96] Lhamo Y., Chugh P.K., Gautam S.R., Tripathi C.D. (2017). Epidemic of Vitamin D Deficiency and Its Management: Awareness among Indian Medical Undergraduates. J. Environ. Public Health.

[97] Vassilopoulou E., Guibas G.V., Papadopoulos N.G. (2022). Mediterranean-Type Diets as a Protective Factor for Asthma and Atopy. Nutrients.

[98] Kim M.J., Kim S.-N., Lee Y.W., Choe Y.B., Ahn K.J. (2016). Vitamin D Status and Efficacy of Vitamin D Supplementation in Atopic Dermatitis: A Systematic Review and Meta-Analysis. Nutrients.

[99] Kim G., Bae J.-H. (2016). Vitamin D and atopic dermatitis: A systematic review and meta-analysis. Nutrition.

[100] Purnamawati S., Indrastuti N., Danarti R., Saefudin T. (2017). The Role of Moisturizers in Addressing Various Kinds of Dermatitis: A Review. Clin. Med. Res..

[101] Painter S.L., Lu W., Schneider J., James R., Shah B. (2020). Drivers of weight loss in a CDC-recognized digital diabetes prevention program. BMJ Open Diabetes Res. Care.

[102] Wollenberg A., Kinberger M., Arents B., Aszodi N., Valle G.A., Barbarot S., Bieber T., Brough H., Pinton P.C., Christen-Zäch S. (2022). European guideline (EuroGuiDerm) on atopic eczema—Part II: Non-systemic treatments and treatment recommendations for special AE patient populations. J. Eur. Acad. Dermatol. Venereol..

[103] Egawa M., Tagami H. (2007). Comparison of the depth profiles of water and water-binding substances in the stratum corneum determined in vivo by Raman spectroscopy between the cheek and volar forearm skin: Effects of age, seasonal changes and artificial forced hydration. Br. J. Dermatol..

[104] Crowther J., Sieg A., Blenkiron P., Marcott C., Matts P., Kaczvinsky J., Rawlings A. (2008). Measuring the effects of topical moisturizers on changes in stratum corneum thickness, water gradients and hydration in vivo. Br. J. Dermatol..

[105] Stratil J.M., Biallas R.L., Burns J., Arnold L., Geffert K., Kunzler A.M., Monsef I., Stadelmaier J., Wabnitz K., Litwin T. (2021). Non-pharmacological measures implemented in the setting of long-term care facilities to prevent SARS-CoV-2 infections and their consequences: A rapid review. Cochrane Database Syst. Rev..

[106] Vyumvuhore R., Tfayli A., Duplan H., Delalleau A., Manfait M., Baillet-Guffroy A. (2013). Effects of atmospheric relative humidity on Stratum Corneum structure at the molecular level: Ex vivo Raman spectroscopy analysis. Analyst.

[107] Boireau-Adamezyk E., Baillet-Guffroy A., Stamatas G.N. (2014). Mobility of Water Molecules in the Stratum Corneum: Effects of Age and Chronic Exposure to the Environment. J. Investig. Dermatol..

[108] Liska D., Mah E., Brisbois T., Barrios P.L., Baker L.B., Spriet L.L. (2019). Narrative Review of Hydration and Selected Health Outcomes in the General Population. Nutrients..

[109] Miyake Y., Yokoyama T., Yura A., Iki M., Shimizu T. (2003). Ecological association of water hardness with prevalence of childhood atopic dermatitis in a Japanese urban area. Environ. Res..

[110] Arnedo-Pena A., Puig-Barberà J., Artero-Civera A., Romeu-Garcia M., Meseguer-Ferrer N., Fenollosa-Amposta C., Vizcaino-Batllés A., Silvestre-Silvester E., Pac-Sa M., Segura-Navas L. (2020). Atopic dermatitis incidence and risk factors in young adults in Castellon (Spain): A prospective cohort study. Allergol. Immunopathol..

[111] Kimata H., Tai H., Nakagawa K., Yokoyama Y., Nakajima H., Ikegami Y. (2002). Improvement of Skin Symptoms and Mineral Imbalance by Drinking Deep Sea Water in Patients with Atopic Eczema/Dermatitis Syndrome (AEDS). Acta Med..

[112] Hataguchi Y., Tai H., Nakajima H., Kimata H. (2005). Drinking deep-sea water restores mineral imbalance in atopic eczema/dermatitis syndrome. Eur. J. Clin. Nutr..

[113] Cacciapuoti S., Luciano M.A., Megna M., Annunziata M.C., Napolitano M., Patruno C., Scala E., Colicchio R., Pagliuca C., Salvatore P. (2020). The Role of Thermal Water in Chronic Skin Diseases Management: A Review of the Literature. J. Clin. Med..

